# Global mental health and trauma: the current evidence and the long road ahead

**DOI:** 10.3402/ejpt.v6.30120

**Published:** 2015-11-19

**Authors:** Marianna Purgato, Miranda Olff

**Affiliations:** 1 Department of Mental Health, Johns Hopkins Bloomberg School of Public Health, Johns Hopkins University, Baltimore, MD, USA; 2 WHO Collaborating Centre for Research and Training in Mental Health and Service Evaluation, Department of Neurological, Biomedical and Movement Sciences, Section of Psychiatry, University of Verona, Verona, Italy, Email: marianna.purgato@univr.it; 3 Department of Psychiatry, Academic Medical Center, University of Amsterdam, Amsterdam, The Netherlands, Arq Psychotrauma Expert Group, Diemen, The Netherlands

In the last few years, a growing amount of evidence highlighted the urgent need for research aimed at assessing mental health status in low- and middle-income countries (LMICs): this revealed a substantial gap between the burden caused by mental disorders and the resources devoted to prevent and treat them (Kakuma et al., 2011). In fact, while more than 80% of the global population lives in LMICs, these countries only manage <20% of the total resources to treat mental illnesses (Patel & Prince, 2010; Saxena, Thornicroft, Knapp, & Whiteford, 2007). As a consequence, more than 75% of people with mental health disorders do not receive care at all, despite substantial role disability (Demyttenaere et al., 2004).

Also research on traumatic stress revealed a predominance of high-income countries (HICs) over LMICs, with a great amount of studies carried out and published by authors affiliated with HICs (Fodor et al., 2014), and the consequent difficulty in transferring evidence results in LMICs contexts (like humanitarian settings).

In response to this call for an evaluation of a “global mental health,” substantial scientific effort was devoted to address the psychological and social needs of populations living in humanitarian settings in LMICs.

This special issue of the *European Journal of Psychotraumatology* collected some of the work that has been done so far in LMICs. Manuscripts address important issues such as the quantification of effects of cumulative trauma exposure in war survivors (Wilker et al., 2015); factors related to psychological distress in orphans living in Tanzania (Hermenau, Eggert, Landolt, & Hecker, 2015); the relationship between trauma exposure and spirit possession (Hecker, Braitmayer, & Van Duijl, 2015); trauma-related mental health problems in humanitarian staff (Strohmeier & Scholte, 2015); and sociotherapy approach developed and implemented in Rwanda (Jansen et al., 2015).

The paper by Wilker et al. (2015) analyzed the role of cumulative trauma exposure as a risk factor for developing posttraumatic stress disorder (PTSD), by assessing reliability and predictive validity of trauma measures in a sample of 227 Ugandan rebel war survivors. Measures showed good reliability and authors concluded that the assessments of event types, together with the additional consideration of event frequencies, may represent a valid and reliable instrument to evaluate cumulative trauma exposure.

Hermenau et al. (2015) undertook a comparison between a group of 89 orphans living in Tanzania and 89 matched non-orphans to explore orphans’ experiences of maltreatment and perceived stigmatization, and to identify possible factors related to psychological distress. In short, findings from this study suggest that, despite no difference being detected in the reported abuse types, orphans experienced more types of neglection, depressive symptoms, PTSD symptoms, and aggressive behavior as compared to non-orphans.

The relation between traumatic experiences and spirit possession in LMICs is analyzed in a systematic review of 21 articles undertaken by Hecker et al. Pathological spirit possession is a phenomenon occurring globally and has particular relevance in LMICs. Reported prevalence rates differed conspicuously depending on cultural background and the particular study populations (Hecker et al., 2015), for example, high rates were found in post-war areas, confirming the connection between traumatic experiences and pathological spirit possession.

The systematic review carried out by Strohmeier and Scholte collected 14 studies focused on trauma-related mental health problems in national humanitarian staff. In line with current literature focused on mental health conditions in LMICs (Barbui & Tansella, 2013), PTSD was the most frequently analyzed outcome, followed by depression and anxiety, while evidence on other disorders like substance use and suicide was particularly scarce (with only one study identified). In general, national staff experienced mental health problems and disorders (prevalence for PTSD, depression, and anxiety) comparably to – if not more than – the control groups (Strohmeier & Scholte, 2015). Even though we have to be cautious before drawing firm conclusions because of the methodological limitations of this review (i.e., included studies present with high level of heterogeneity), at least two messages are of importance: mental health problems need to be considered not only in the general population but also in humanitarian staff, as staff are exposed to same risk factors. Second, most frequently mental health researchers have focused on PTSD, but other mental health problems (e.g., suicide risk and substance abuse) are similarly relevant in LMICs and there is an urgent need for attention to these conditions.

Jansen et al. (2015) propose a critical reflection on the evidence-based approach for mental health interventions, highlighting the preponderance of psychiatric approaches in international guidelines on mental health conditions. In particular, authors stress the risk of medicalization of social problems and present an example of a sociotherapy approach developed in Rwanda to show how communities of support may play a role in promoting mental health and psychosocial well-being.

This cluster of manuscripts approaches global mental health using different study designs, which report both the patients and staff perspectives. Aware of the complexity and extent of the topic, we believe that these papers will contribute to the scientific debate in this field and represent a relevant piece of work in the reasoning on how to orient further research pathways. This become even more important in this historical time frame, where populations living in humanitarian conditions and affected by different types of trauma – including refugees inside and outside Europe (Turner, 2015), asylum seekers, people affected by natural disasters (those living in Nepal are only one example) – are at the center of the attention of clinicians, researchers, policy makers, and media.

*Marianna Purgato* Department of Mental Health Johns Hopkins Bloomberg School of Public Health Johns Hopkins University, Baltimore, MD, USA WHO Collaborating Centre for Research and Training in Mental Health and Service Evaluation Department of Neurological, Biomedical and Movement Sciences Section of Psychiatry, University of Verona, Verona, Italy Email: marianna.purgato@univr.it *Miranda Olff* Department of Psychiatry, Academic Medical Center University of Amsterdam, Amsterdam, The Netherlands Arq Psychotrauma Expert Group, Diemen The Netherlands
